# The Impact of Antibiotic Usage Guidelines, Developed and Disseminated through Internet, on the Knowledge, Attitude and Prescribing Habits of Orthokeratology Contact Lens Practitioners in China

**DOI:** 10.3390/antibiotics11020179

**Published:** 2022-01-29

**Authors:** Zhi Chen, Jifang Wang, Jun Jiang, Bi Yang, Pauline Cho

**Affiliations:** 1 Eye Institute and Department of Ophthalmology, Eye and ENT Hospital, Fudan University, Shanghai 200031, China; carol1979@yeah.net; 2 NHC Key Laboratory of Myopia, Fudan University, Shanghai 200031, China; 3 Key Laboratory of Myopia, Chinese Academy of Medical Sciences, Shanghai 200031, China; 4 Department of Contact Lens Clinic, The Eye Hospital of Wenzhou Medical University, Wenzhou 325027, China; jjhsj@hotmail.com; 5 Department of Optometry and Vision Sciences, West China School of Medicine, Sichuan University, Chengdu 610017, China; yangbi19830418@126.com; 6 School of Optometry, The Hong Kong Polytechnic University, Hong Kong SAR, China; pauline.cho@connect.polyu.hk

**Keywords:** misuse of antibiotics, orthokeratology, contact lens, microbial keratitis, questionnaire

## Abstract

It has been previously reported that the improper prescribing of antibiotic eye drops is common among orthokeratology (ortho-k) practitioners. Guidelines have since been developed and disseminated to improve their understanding and implementation of antibiotic prescriptions. This study aimed to investigate the influence of these guidelines on the knowledge, attitude, and prescribing habits of ortho-k practitioners by means of a questionnaire, which was administered nationwide via an official online account to eye care practitioners (ECPs) involved in ortho-k lens fitting, 548 of whom completed the survey. Differences in characteristics before and after the dissemination of the guidelines and between the groups were explored using χ^2^ tests. The relationship between prescribing habits and demographics was analyzed using stepwise logistic regression models. The implementation of the guidelines significantly improved the overall prescribing habits of ECPs (*p* < 0.001), especially for prophylactic antibiotic use before and after ortho-k lens wear (*p* < 0.001). Most ECPs who prescribed antibiotics properly displayed significantly better knowledge of correct antibiotic use, which in turn affected the compliance in their ortho-k patients (*p* < 0.001). The ECPs’ occupations (professionals other than ophthalmologists and optometrists, including nurses and opticians), clinical setting (distributor fitting centers), and age (younger than 25 years) were risk factors for the misuse of antibiotics. Although the implementation of the antibiotic guidelines significantly improved overall prescribing habits, some practitioners’ prescribing behavior still needs improvement. A limitation of this study was that all questions were mandatory, requiring ECPs to recall information, and therefore was subjected to selection and recall bias.

## 1. Introduction

Orthokeratology (ortho-k) employs specially designed rigid gas-permeable contact lenses, worn overnight to correct myopic refractive error during the day through temporary molding of the cornea [1]. The mechanism underlying the corneal reshaping process mainly involves producing hydraulic force beneath the lens, leading to the redistribution of corneal epithelial cells [2], resulting in central flattening and mid-peripheral steepening [3]. In addition to this vision-correcting function, ortho-k has been shown to slow myopia progression in children and juveniles. Studies have shown its myopia control efficacy, based on the percentage of axial elongation reduction, to range from 36% to 46% [4,5,6,7].

In the last decade, ortho-k has become the most accessible and popular myopia control modality in China, with over 1.5 million users in 2016 [8]. The number of eye care practitioners (ECPs) also increased dramatically to meet the huge demand for ortho-k. The International Academy of Orthokeratology Asia (IAOA) trained over 10,000 ECPs and issued over 1700 memberships between 2012 and 2016 in Mainland China [8]. Ophthalmologists and optometrists holding a university degree or diploma working in the same clinical setting with ophthalmologists are considered to be the best qualified professionals to fit ortho-k lenses and to prescribe antibiotic eye drops associated with ortho-k lens wear. Other professionals, such as nurses and opticians, are occasionally involved in ortho-k lens practice, most frequently in private clinics and distributor fitting centers, under the supervision of ophthalmologists. In the current study, these professionals are all listed as ECPs.

The safety of overnight contact lens therapy is an important concern for ECPs, especially when this treatment targets children and juveniles. As overnight ortho-k is associated with reduced tear exchange and oxygen tension at the ocular surface, it is suggested that microorganisms build up during sleep and increase the likelihood of microbial keratitis (MK) [9]. Although the overall safety of overnight ortho-k is reported to be good, with the incidence of MK being similar to that of other overnight or extended wear contact lens modalities [10], its consequences can be sight threatening. Typically, ortho-k-related MK occurs 5 to 15 months after the commencement of treatment [10]. Early diagnosis and treatment of MK are extremely important for prognosis, and correct antibiotic use is essential for the effective management of the infection. The most commonly prescribed antibiotic eye drops, unlike the long-favored triple-agent formulation of polymyxin, neomycin, and bacitracin, contain either 0.3% tobramycin (aminoglycoside) or 0.5% levofloxacin (fluoroquinolone).

A recent survey used a questionnaire to explore the antibiotic (eye drops) prescribing habits of ECPs who fit ortho-k contact lenses in Mainland China. The results indicated that the misuse or improper use of antibiotic eye drops is common among ECPs [11]. Therefore, guidelines based on best practice and taking into account deficiencies and errors identified from the survey, were developed and issued to ortho-k ECPs nationwide via an official online account [11]. The reinforcement of the importance of proper antibiotic use in ortho-k practice lasted for six months, mainly through online education.

As these guidelines were promulgated for six months, the purpose of this study was to investigate their effect on knowledge, attitude, and behavioral change in antibiotic use among ortho-k ECPs in China. Further issues identified from this survey may provide insights into a more efficient means of practitioner education, not only for ECPs, but also for other medical practitioners.

## 2. Results

**Figure 1:**
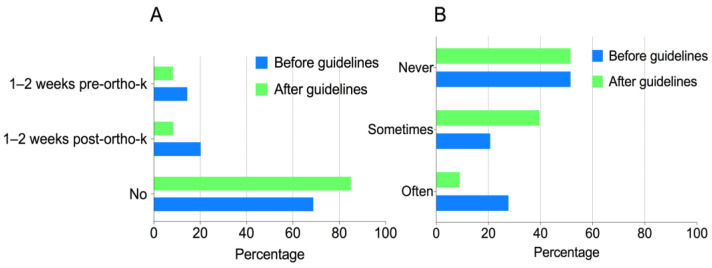
Average frequencies (%), before and after the administration of guidelines, of (**A**) prescription of prophylactic antibiotic eye drops before and after the commencement of ortho-k treatment; (**B**) use of antibiotic eye drops for wetting fluorescein strips during ortho-k lens fitting.

**Figure 2:**
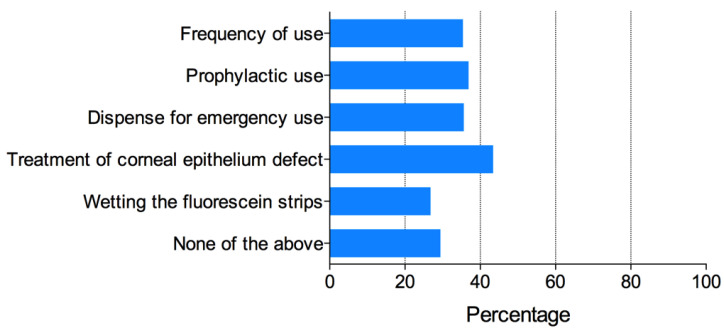
Which aspects of your practice did the article and guidelines change, with respect to the use of antibiotic eye drops in orthokeratology therapy?

**Figure 3:**
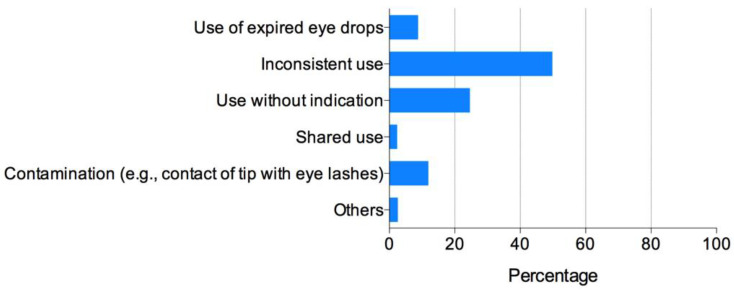
What is the most common misuse of antibiotic eye drops in your orthokeratology patients?

Over 4000 subscribers of the IAOA official online account received the invitation to complete the questionnaire. A total of548 recipients completed and returned the questionnaire. The respondents were composed of ophthalmologists (20.9%), optometrists (65.5%), and others (13.6%). The details of the respondents are shown in Table 1. Respondents’ knowledge, attitude, and behaviors regarding antibiotic use are shown in Table 2 and Figure 1, Figure 2 and Figure 3.

The implementation of the guidelines on the use of antibiotics significantly improved the overall behavior of the ECPs. A total of 39.2% of the respondents of the current study presented the appropriate use of antibiotic eye drops in all three aspects surveyed, compared to 25.9% in the previous study (*p* < 0.001) [11]. The number of antibiotic eye drop prescriptions (based on estimation of purchases) decreased in the preceding six months, with a higher proportion of the respondents reporting less frequent use (i.e., in 0–20% of their patients) than before (*p* = 0.027).A significantly higher proportion of the respondents (84.5%) did not use antibiotics prophylactically in ortho-k patients, compared to the previous survey (67.6%) (*p* < 0.001) (Figure 1A) [11]. A total of 48.5% of the respondents used antibiotic eye drops to wet fluorescein strips, which did not differ from the 48.5% reported in the previous survey. However, when considering the frequency of this misuse, considerably fewer respondents reported “always” or “often” using antibiotic eye drops to wet fluorescein strips (9.0%), as compared to the previous survey (27.7%) (*p* < 0.001) (Figure 1B) [11]. A total of 36.1% of the respondents did not give clear written instructions along with the dispensing of antibiotics to patients, showing no difference compared to the previous survey (40.7%) (*p* = 0.132).

A total of 215 of the respondents (39.2%) used antibiotic eye drops properly in all three aspects surveyed. Their overall knowledge of antibiotic use was significantly better than their counterparts who misused antibiotics in at least one of the behavior-related questions. For example, for the question “antibiotic eye drops may be used prophylactically before or after commencement of ortho-k treatment to prevent corneal infection”, 90.6% of the respondents who used antibiotics properly “disagreed” or “strongly disagreed” with this statement, compared to 75.7% of their counterparts (*p* < 0.001). Additionally, for the question “avoid dispensing antibiotic eye drops to patients for emergency use (if unavoidable, dispense together with clear written instructions)”, 75.3% of the respondents who used antibiotics properly “agreed” or “strongly agreed” with this statement, compared to 46.4% of their counterparts (*p* < 0.001).

The overall attitude towards the proper use of antibiotics was positive among all the respondents. A total of 89.3% of the respondents “agreed” or “strongly agreed” that it is very important to properly use antibiotic eye drops in ortho-k treatment. The vast majority (96.9%) of the respondents “agreed” or “strongly agreed” that the article and the guidelines were useful. A total of 99.1% of all the respondents “agreed” or “strongly agreed” that they were more careful when considering the use of antibiotics in ortho-k treatment after reading the guidelines. For the question “in what aspects did the article and the guidelines change my practice”, the greatest change in behavior was “in the treatment of corneal epithelium defects after ortho-k treatment” (43.4%), and the least change was “in wetting the fluorescein strip using antibiotic eye drops during ortho-k lens fitting” (26.8%) (Figure 2).

The most common misuse of antibiotic eye drops by the patients was “applying eye drops irregularly (period of use or frequency)” (49.8%), followed by “using when not indicated (e.g., allergic conjunctivitis or dry eye)” (24.6%) and “contaminating eye drops via contact of the bottle tip with the eyelashes” (11.9%) (Figure 3). The respondents who used antibiotics properly reported significantly fewer of their patients misusing antibiotics than the ECPs who did not (*p* < 0.001).

Overall, occupation, age, and clinical setting all significantly affected the likelihood of respondents’ using antibiotics correctly (Table 3). Significantly higher chances of misusing antibiotics, especially using antibiotic eye drops to wet fluorescein strips, and dispensing antibiotics to patients for emergency use without giving clear written instructions were found among professionals (e.g., nurses and opticians) other than ophthalmologists and optometrists (both *p* < 0.05). Compared to younger respondents (<25 years), older ECPs appeared to be more likely to use antibiotics correctly. Younger respondents (<25 years) were more likely to wet the fluorescein strip with antibiotic eye drops and to dispense antibiotics to patients for emergency use without giving clear written instructions (both *p* < 0.05). Compared to other clinical settings, ECPs in the distributor fitting centers were more likely to use antibiotics improperly, including wetting the fluorescein strip with antibiotic eye drops and dispensing antibiotics to patients for emergency use without giving clear written instructions (both *p* < 0.05). However, they were less likely to use antibiotics prophylactically before or after the commencement of ortho-k treatment (*p* < 0.05).

## 3. Discussion

This study found that, compared to the previous survey, before the development and dissemination of guidelines on the use of antibiotic eye drops, the overall behavior of the respondents had improved significantly since the implementation of the guidelines six months earlier, although the prescribing habits of some ECPs required further improvement.

The greatest change occurred in the prophylactic use of antibiotics before or after ortho-k treatment, as evidenced by a higher proportion (84.5%) of respondents not using antibiotics prophylactically, as compared to the previous survey (67.6%), and also by a decreased number of antibiotic prescriptions in the preceding six months. The most common reasons for using prophylactic antibiotics among ECPs, as reflected by the current survey were: 1. to prevent infection in case of corneal injury during the early phase of ortho-k lens wear; 2. to reduce the discomfort of ortho-k lens wear; 3. to reduce ocular discharge during the early adaptation period of ortho-k; 4. to prevent infection in all sorts of contact lens wear. Since the eyes of healthy young ortho-k patients do not usually carry a heavy burden of microorganisms, there is no evidence that antibiotic eye drops can prevent contact lens-related MK. Notably, ortho-k-induced MK typically occurs five to 15 months after the commencement of lens wear [10], and is most likely to be induced by improper lens care, such as inadequate hand washing, rinsing, storing contact lenses in tap water, or poor hygiene with lens cases and suction holders [12,13]. In addition, symptoms, including foreign body sensation, and clinical signs, such as increased discharge in the early phase of ortho-k lens wear, can be completely normal, so antibiotics for these problems are not only useless, but also add to the risk of bacterial resistance to antibiotics.

Surprisingly, the implementation of the guidelines barely had any effect on the ECPs’ habits of prescribing antibiotics to ortho-k patients for use at their own discretion during emergencies. A total of 36.1% of the respondents did not give clear written instructions when dispensing antibiotics to patients. The respondent’s occupation (most frequent in “others”, such as nurse and optician), the ECP’s age (most frequent in those <25 years), and clinical setting (most frequent in distributor fitting centers) were identified as risk factors for this behavior. The most common reasons include: 1. patients live far away or overseas, and it is inconvenient to come to the clinic during an emergency; 2. patients have a heavy academic burden and are not compliant with routine follow-up visits; 3. patients may suffer seasonal allergic conjunctivitis and may need antibiotics “just in case”; 4. patients ask for antibiotics. It is evidenced by our study that the most common misuse of antibiotics among ortho-k patients is to use them irregularly (e.g., period of use or frequency) (49.8%) and to use them when not indicated (e.g., allergic conjunctivitis or dry eye) (24.6%). While it can be argued that medical resources are unevenly distributed across different regions in China and that patients may not have access to their ECP during an emergency, it is doubtful that dispensing antibiotics to patients is justified or ethical. An emergency contact number should be provided to patients along with clear instructions as to when to call [14]. For patients with poor compliance with follow-up visits, there is no reason to believe that they can use antibiotics correctly when indicated and, considering the potential risk arising from non-compliance with treatment, they are probably not suitable candidates for ortho-k treatment. Currently, there are several novel non-contact lens myopia control modalities other than ortho-k, including specially designed spectacles [15,16] and low-concentration atropine [17,18], which could be considered when compliance is an issue for patients.

One of the disturbing behaviors that still needs urgent attention is the use of antibiotic eye drops to wet fluorescein strips during ortho-k lens fitting. It is worrisome that no change has been observed in the proportion of ECPs conducting this practice since the administration of the guidelines, although significantly fewer respondents reported that they “always” or “often” use antibiotic eye drops to wet fluorescein strips (9.0%), as compared to the previous survey (27.7%). In concordance with this finding is that the fewest respondents reported a change in this behavior when responding to the question “In what aspects did the article and the guidelines change your practice”. The respondent’s occupation (most frequent in “others”, such as nurse and optician), the ECP’s age (most frequent in <25 years) and clinical setting (most frequent in distributor fitting centers) were the risk factors identified for this behavior. The most common reasons for this behavior are that: 1. antibiotic eye drops are easily accessible in the clinic; 2. no other solutions, such as saline solutions, are available in the clinic; 3. antibiotic eye drops may decrease the irritation and prevent infection during ortho-k lens fitting. These results indicated that the clinical setting plays a major role in the regulation of antibiotic use in ortho-k lens fitting. Hospitals and clinics should restrict the use of antibiotics, provide ortho-k ECPs with antibiotic stewardship programs (especially for younger ECPs and ECPs identified as other than ophthalmologists and optometrists working in distributor fitting centers), and, most importantly, provide non-preserved saline solution instead of antibiotic eye drops to wet fluorescein strips during ortho-k lens fitting. Trial lens disinfection should also be emphasized in clinical settings to address ECPs’ concerns about cross-infection among ortho-k patients.

Generally, the overall attitude towards proper antibiotic use was positive. Therefore, the misuse of antibiotics by some respondents cannot be solely attributable to their indifference to the guidelines. The guidelines have been distributed on various online platforms and read by ECPs over 5000 times. Since the correlation between knowledge and behavior was found to be strong in this study, we are tempted to conclude that the efficiency of education mainly through online social media was insufficient. Reinforcement of guidelines in off-line conferences and continuing education programs are also suggested. Interestingly, it was noted that the respondents who used antibiotics properly reported fewer of their patients misusing antibiotics than the ECPs who did not. This finding indicated that ECPs’ knowledge about antibiotics might impact their behavior, which in turn influences their patients’ compliance with antibiotic use.

One important issue identified in the current study that needs urgent attention is that the overall antibiotic misuse is most common among professionals other than ophthalmologists and optometrists, i.e., nurses and opticians. They are less likely to receive full-term medical training or optometric education, not to mention fitting specialty contact lenses such as ortho-k. Therefore, not only should the guidelines on antibiotic use be delivered to them in a timely manner, but regulations should also be reinforced to limit their prescription of this specialized therapy.

One of the limitations of this study was that the two surveys were not conducted on the same cohort, since the first one was performed anonymously [11]. Therefore, the data collected could not be used to directly compare the behaviors before and after the administration of guidelines in individuals, but rather, in a group of practitioners. However, the respondents were evenly distributed across all clinical settings, indicating that the sample was representative in terms of medical practice. As with all self-administered questionnaires, there is always a question of the truthfulness of the responses. A survey consisting of all mandatory questions may contribute to a selection bias in the participants of the survey. Additionally, the surveys were based on impressions or perceptions of how the prescriber uses antibiotics and did not always correspond to prescribing or dispensing data with respect to the best practices in antimicrobial use. However, even if a respondent did not answer all questions accurately, just responding to the questions would result in a reminder of the correct procedures. As such, surveys can act as a tool for the reinforcement of good practice.

## 4. Materials and Methods

### 4.1. Preparation of the Questionnaire

A questionnaire (see Supplementary Material S1), comprising 21 questions (16 related to the knowledge, attitude, and behavior in antibiotic prescription and five related to demographics), was prepared and reviewed by five contact lens specialists from four top-ranking national ophthalmology departments. The questionnaires were prepared initially in English, translated into Chinese, and then translated back into English to check for accuracy. The respondents were requested to complete the questions in the Chinese version. All the questions in this survey were mandatory. The questionnaire was then sent to 35 ECPs involved in ortho-k lens treatment nationwide (not included in the final analysis) for validation and to calculate the internal consistency of the questionnaire. The Cronbach’s alpha coefficient was 0.75, which indicated an acceptable level of the questionnaire’s reliability. The three key questions regarding habitual use of antibiotic eye drops were:

- Do you prescribe prophylactic antibiotic eye drops before or after fitting ortho-k?
- Do you dispense antibiotic eye drops to patients for emergency use (at patients’ discretion)?
- Do you use antibiotic eye drops to wet fluorescein strips during ortho-k lens fitting?

### 4.2. Definition of Proper Antibiotic Use

To further determine which group of ECPs was more likely to misuse or overuse antibiotics, and whether the circulated guidelines had had any effect on the habitual use of antibiotics by ECPs, options for the above-mentioned questions were categorized based on the definitions of proper use of antibiotics below:

- Do not use prophylactic antibiotic eye drops before or after fitting ortho-k lenses;
- Do not dispense antibiotic eye drops to patients for emergency use (when essential, dispense to patients along with written instructions);
- Do not use antibiotic eye drops to wet fluorescein strips during ortho-k lens fitting.

### 4.3. Distribution of Questionnaires

The questionnaires were distributed through the official online account of the International Academy of Orthokeratology Asia (IAOA), which has over 4000 members listed as ortho-k practitioners throughout China. The survey was conducted over June and July 2020. The inclusion criteria comprised ECPs, including ophthalmologists, optometrists, and other professionals, such as nurses and opticians, who had been involved in ortho-k lens fitting and patient care. 

### 4.4. Statistical Analyses

Data concerning prescribing habits (16 questions) and demographics of the ECPs (five questions) were expressed as percentages. Data of the three key prescribing habits were compared to those of the previous study using χ^2^ tests. Using the definition of proper antibiotic use, ECPs were further divided into groups, and the differences between the groups were tested using χ^2^ tests for categorical variables. Stepwise logistic regression models were used to assess the relationship between prescribing habits and demographics. Two-sided values of *p* < 0.05 were deemed statistically significant. All statistical analyses were performed using software R (version 4.0.5).

## 5. Conclusions

The current study revealed that the overall behavior of the ortho-k ECPs significantly improved after the nationwide implementation of guidelines on antibiotic use, although some ECPs’ prescribing habits require further improvement. Since the ECPs’ behavior was significantly affected by their knowledge, the effective delivery of education is the key to success: it should not only be delivered online, but also off-line to enhance its efficacy. Clinical settings, such as hospitals and clinics, can also play an important role by providing ECPs with non-preserved saline solution to replace antibiotic eye drops in ortho-k lens fitting, and by introducing an antibiotic stewardship program, especially for younger ECPs. Rigorous clinical governance measures, particularly at the distributor fitting centers, are strongly recommended with regard to ortho-k practice and antibiotic use.

## Figures and Tables

**Table 1:** Demographics of respondents (*n* = 548).

	Frequency (%)
1. Occupation Ophthalmologist Corneal specialist Refractive surgeon Medical doctor conducting optometry (non-surgical)	114 (20.9) 7 (1.3) 8 (1.5) 99 (18.1)
Optometrist (degree or diploma)	359 (65.5)
Other (nurse, optician, etc.)	75 (13.6)
2. Clinical setting	
General hospital	142 (25.9)
Ophthalmic specialty hospital	120 (21.9)
Private optometry clinic	144 (26.3)
Ortho-k distributor fitting center	142 (25.9)
3. Level of practice	
Provincial level	111 (20.3)
Municipal level	307 (56.0)
County level	55 (10.0)
Others	75 (13.7)
4. Age (years)	
<25	77 (14.1)
25–30	174 (31.8)
31–35	124 (22.6)
36–40	76 (13.9)
>40	97 (17.7)
5. Sex	
Male	143 (26.1)
Female	405 (73.9)

**Table 2:** Respondents’ knowledge and attitudes towards antibiotic eye drop use in orthokeratology practice (*n* = 548) (%).

Do You Agree with the Statement	Strongly Agree	Agree	Not Sure	Disagree	Strongly Disagree
1. “antibiotic eye drops may be used prophylactically before or after commencement of treatment to prevent corneal infection”?	7 (1.3)	42 (7.7)	57 (10.4)	321 (58.6)	121 (22.0)
2. “when bacterial keratitis is suspected, patients do not have to stop lens wear, but re-enforcement of lens care routines and use of broad-spectrum antibiotics is necessary”?	4 (0.7)	16 (2.9)	24 (4.4)	168 (30.7)	336 (61.3)
3. “avoid dispensing antibiotic eye drops to patients for emergency use (if unavoidable, dispense together with clear written instructions)”?	98 (17.9)	209 (38.1)	41 (7.5)	183 (33.4)	17 (3.1)
4. “it is very important to properly use antibiotic eye drops”?	361 (65.9)	128 (23.4)	26 (4.7)	28 (5.1)	5 (0.9)
5. “the article and the guidelines are useful”?	342 (62.4)	189 (34.5)	12 (2.2)	3 (0.5)	2 (0.4)
6. “I will consider more carefully when using antibiotics after reading the guidelines”?	375 (68.4)	168 (30.7)	4 (0.7)	1 (0.2)	0 (0)

**Table 3:** The results of logistic regression models of respondents’ demographics on overall proper antibiotic eye drop use.

		OR (95% CI)	*p*
Occupation	Ophthalmologist	Referent	
	Optometrist (degree or diploma)	0.68 (0.44–1.05)	0.08
	Other (nurse, optician, etc.)	0.23 (0.11–0.48)	<0.001
Clinical setting	Private optometry clinic	Referent	
	Ophthalmic specialty hospital	1.08 (0.66–1.78)	0.75
	General hospital	1.02 (0.64–1.65)	0.92
	Distributor fitting center	0.27 (0.15–0.48)	<0.001
Practice level	Provincial	Referent	
	Municipal	0.77 (0.49–1.22)	0.27
	County	1.01 (0.52–1.99)	0.97
	Others	1.12 (0.61–2.05)	0.72
Age	<25	Referent	
	25–30	2.94 (1.5–5.74)	<0.001
	31–35	2.61 (1.3–5.27)	0.01
	36–40	2.87 (1.35–6.12)	0.01
	>40	2.54 (1.22–5.26)	0.01
Sex	Male	Referent	
	Female	1.46 (0.96–2.22)	0.08

## Data Availability

Data are available upon request.

## Supplementary Materials

The following supporting information can be downloaded at: https://www.mdpi.com/article/10.3390/antibiotics11020179/s1, File S1: Questionnaire.
