# Supplementary material for: The Impact of Antibiotic Usage Guidelines, Developed and Disseminated through Internet, on the Knowledge, Attitude and Prescribing Habits of Orthokeratology Contact Lens Practitioners in China
Source: Antibiotics (Basel). 2022 Jan 29;11(2):179. doi: 10.3390/antibiotics11020179 (PMC8868172; doi:10.3390/antibiotics11020179)
Supplement: Supplementary file 1 [file antibiotics-11-00179-s001.zip › antibiotics-1484070-supplementary.pdf]

The goal of this questionnaire is to understand the influence of the provision of guidelines on antibiotics use in orthokeratology practice in China. Data collected may be used for publication and/or academic report. Please answer honestly.

Knowledge dimensions

1. Antibiotic eye drops may be used prophylactically BEFORE or AFTER commencement of ortho-k treatment to prevent corneal infection

   □ strongly agree □ agree □ not sure □ disagree □ strongly disagree

2. When bacterial keratitis is suspected during ortho-k treatment, patients do not have to stop lens wear, but re-enforcement of lens care routines and use of broad-spectrum antibiotics is necessary

   □ strongly agree □ agree □ not sure □ disagree □ strongly disagree

3. Avoid dispensing antibiotic eye drops to patients for emergency use (if unavoidable, dispense together with clear written instructions)

   □ strongly agree □ agree □ not sure □ disagree □ strongly disagree

Attitude dimensions

4. I think it is very important to properly use antibiotic eye drops in ortho-k treatment

   □ strongly agree □ agree □ not sure □ disagree □ strongly disagree

5. I find the article and the guidelines useful

   □ strongly agree □ agree □ not sure □ disagree □ strongly disagree

6. I will consider more carefully when using antibiotics in ortho-k treatment after reading the guidelines

   □ strongly agree □ agree □ not sure □ disagree □ strongly disagree

7. In what aspects did the article and the guidelines change my practice? (Please select all that apply)

☐ In the frequency of antibiotic eye drops use in ortho-k patients

☐ In the prophylactic use of antibiotic eye drops in ortho-k patients

☐ In dispensing antibiotic eye drops to ortho-k patients for emergency use

☐ In the treatment of corneal epithelium defect after ortho-k treatment

☐ In wetting the fluorescein strip using antibiotic eye drops during ortho-k lens fitting

☐ None of the above has changed

Practice dimension

8. What is the average frequency of antibiotic eye drops use in your ortho-k patients (%) in the last 6 month?

☐ 0-20 ☐ 21-40 ☐ 41-60 ☐ 61-80 ☐ 81-100

9. What was the % BEFORE you read the guidelines ____________________

☐ 0-20 ☐ 21-40 ☐ 41-60 ☐ 61-80 ☐ 81-100

10. Do you prescribe prophylactic antibiotic eye drops before and after the commencement of ortho-k treatment?

☐ Yes (multiple choices allowed)

☐ Within one week BEFORE commencing ortho-k

☐ More than one week BEFORE commencing ortho-k

☐ Within one week AFTER commencing ortho-k

☐ More than one week AFTER commencing ortho-k

☐ No

11. If the answer is yes, please give reasons ____________________________

12. Do you dispense antibiotic eye drops to patients for emergency use (at patients' discretion)? (single choice only)

 Yes

- No instructions are given on how to use the drug
- Only oral instructions are given on how to use the drug
- Only written instructions are given on how to use the drug
- Both oral and written instructions are given on how to use the drug

 No

13. If the answer is yes, please give reasons ______________________________

14. Do you use antibiotic eye drops to wet the fluorescein strip during ortho-k lens fitting?

 Always Often Sometimes Occasionally Never

15. If the answer is yes, please give reasons ______________________________

16. The <u>most common misuse</u> of antibiotic eye drops in your patients are

 Using overdue eye drops

 Applying eye drops irregularly (period of use or frequency)

 Using when not indicated (e.g., allergic conjunctivitis or dry eye)

 Sharing eye drops with others

 Contaminating eye drops via contact of the bottle tip with the eyelashes

Demographics

17. What is your occupation? (single choice only)

 Ophthalmologist

- Corneal specialist
- Refractive surgeon
- Medical doctors conducting optometry (non-surgical)

□ Optometrist (degree or diploma)

□ Other (nurse, optician, etc.)

18. What is your clinical setting? (single choice only)

 □ General hospital

 □ Ophthalmic specialty hospital

 □ Private optometry clinic

 □ Ortho-k distributor's fitting center

19. What is the level (in the medical service system) of your practice? (single choice only)

□ Provincial level

□ Municipal level

□ County level

□ Others

20. What is your age? (single choice only)

 □ <25 years

 □ 25-30 years

 □ 31-35 years

 □ 36-40 years

 □ >40 years

21. What is your sex? (single choice only)

 □ Male

 □ Female

22. Your phone number? (optional, for interview purposes ONLY)

____

Thank you for completing this questionnaire !
